# Comparison of quenching and extraction methodologies for metabolome analysis of *Lactobacillus plantarum*

**DOI:** 10.1186/1475-2859-6-27

**Published:** 2007-08-20

**Authors:** Magda Faijes, Astrid E Mars, Eddy J Smid

**Affiliations:** 1 TI Food & Nutrition, PO Box 557, 6700 AN Wageningen, The Netherlands; 2 Wageningen UR, Agrotechnology and Food Sciences Group, PO Box 17, 6700 AA Wageningen, The Netherlands; 3 NIZO food research, PO Box 20, 6710 BA, Ede, The Netherlands; 4 Institut Químic de Sarrià, Universitat Ramon Llull, 08017 Barcelona, Spain

## Abstract

**Background:**

A reliable quenching and metabolite extraction method has been developed for *Lactobacillus plantarum*. The energy charge value was used as a critical indicator for fixation of metabolism.

**Results:**

Four different aqueous quenching solutions, all containing 60% of methanol, were compared for their efficiency. Only the solutions containing either 70 mM HEPES or 0.85% (w/v) ammonium carbonate (pH 5.5) caused less than 10% cell leakage and the energy charge of the quenched cells was high, indicating rapid inactivation of the metabolism.

The efficiency of extraction of intracellular metabolites from cell cultures depends on the extraction methods, and is expected to vary between micro-organisms. For *L. plantarum*, we have compared five different extraction methodologies based on (i) cold methanol, (ii) perchloric acid, (iii) boiling ethanol, (iv) chloroform/methanol (1:1) and (v) chloroform/water (1:1). Quantification of representative intracellular metabolites showed that the best extraction efficiencies were achieved with cold methanol, boiling ethanol and perchloric acid.

**Conclusion:**

The ammonium carbonate solution was selected as the most suitable quenching buffer for metabolomics studies in *L. plantarum *because (i) leakage is minimal, (ii) the energy charge indicates good fixation of metabolism, and (iii) all components are easily removed during freeze-drying. A modified procedure based on cold methanol extraction combined good extractability with mild extraction conditions and high enzymatic inactivation. These features make the combination of these quenching and extraction protocols very suitable for metabolomics studies with *L. plantarum*.

## Background

The metabolome of a micro-organism is a reflection of its metabolic state and therefore contains information about the biological processes that are active under particular growth conditions. The *in vivo *determination of metabolite concentrations in cell cultures is possible using NMR, but the application is limited to specific groups of metabolites (i.e. phosphorous containing metabolites) or requires the use of stable isotope labelled substrates [1-6]. The major limitation of NMR analysis is the relatively low sensitivity.

When metabolites are analysed in *in vitro *samples, it is essential that the sample reflects the biological status of interest. Representative samples for metabolome analysis can only be taken when inactivation of the metabolism is rapid compared to the metabolic reaction rates. For growing microorganisms, the turnover of intracellular metabolites can be extremely fast. Cytosolic glucose in *Saccharomyces cerevisiae *is converted at a rate of approximately 1 mM s^-1 ^[7], while ATP and ADP turnover rates are in the range of 1.5 to 2.0 mM s^-1 ^[8]. Consequently, instantaneous fixation of the metabolism during sampling is essential.

Instantaneous inactivation of metabolism is often achieved by rapidly decreasing the culture temperature to values far below 0°C. When separation of intra- and extracellular metabolites is needed, it is important that cells retain their integrity. Bolten et al. [9] clearly demonstrated for a range of different micro-organisms that methanol quenching with subsequent separation of cells and supernatant causes severe leakage of metabolites and consequently underestimation of the intracellular levels.

Quenching of the culture in an aqueous methanol solution at temperatures of -40°C or -50°C has become a standard procedure [7]. It is widely used for *Escherichia coli *and *S. cerevisiae *[7,10-13]. However, it has never been demonstrated that the metabolism of the microorganisms was inactivated sufficiently fast. Instead, the assumption was made that the metabolism was adequately fixed due the use of rapid sampling techniques or the presence of intermediates of glycolysis in the sample [7,10-13]. In this study, we applied the energy charge parameter (EC) as an indicator to determine the inactivation of cell metabolism. This parameter describes the relationship between ATP, ADP and AMP in the cell, and therefore indicates the energy status of a biological system [14]. Energy charge values between 0.8–0.9 have been reported for growing cells of, for example, *Bacillus subtilis*, *S. cerevisiae *or *Lactococcus lactis *[15-18]. This value drops below 0.18 within one minute after the removal of the growth substrate [18]. When the energy charge value was calculated from literature data, results are controversial. The standard procedure using cold aqueous methanol solution with *S. cerevisiae *[7] resulted in cells in which the amount of adenylated nucleotides corresponded to an energy charge value below 0.2, indicating that the cells were energetically starving and did not represent the growing cells of the cultures. Besides, this methodology has also been used to quench the metabolism of *E. coli *[12], which resulted in an energy charge value in the same range. These studies show that the metabolite profiles obtained with these methods do not reflect the metabolome of growing cells.

After quenching and harvesting microbial cell cultures, the intracellular metabolites need to be extracted from the cell pellet. Ideally, the method should extract all metabolites in a non-selective and reproducible way and should inhibit all chemical and enzymatic conversions. Different extraction procedures have been described, but none of them meets all of these criteria. Most extraction methods are designed for specific classes of metabolites, and often introduce harsh conditions like extreme pH values that lead to degradation of certain metabolites [7,19,20]. Alternatives are neutral extraction agents like chloroform [7], boiling ethanol or methanol [11,19,21]. However, low polarity and solubility of metabolites in chloroform and side-reactions or loss of metabolites due to high temperature are intrinsic disadvantages of these extraction agents [22]. Ideally, an efficient extraction method should be applicable for a variety of different micro-organisms, but the susceptibility to lytic conditions is known to differ between species.

Quenching and extraction procedures need to be validated for each microorganism of interest. In this study, we tested different quenching and extraction procedures for their suitability for *Lactobacillus plantarum*, and determined the energy charge to demonstrate rapid inactivation of the metabolism. *L. plantarum *is a Gram-positive bacterium that occurs in a large variety of ecological niches, including the human gastrointestinal tract, in which it may confer various health benefits for the consumer upon ingestion [23]. The genome sequence of *L. plantarum *WCFS1 has been published [24], and the strain is currently being subjected to functional genomics research [25-28].

## Results and Discussion

### Quenching of metabolism

To develop a method for representative sampling of the intracellular metabolite pool of a growing culture of *L. plantarum*, different quenching solutions, applied at -40°C, were evaluated for two requirements: (i) the ability to both retain the integrity of cell envelope and (ii) the immediate inactivation of metabolism.

Four different quenching solutions were used in this study. The first one is 60% MeOH, which was used for *S. cerevisiae *and does not contain any disturbing components that could interfere with posterior metabolite analyses [7,11]. The absence of cell lysis with this solution was proven for *S. cerevisiae*. However, some microbial species show lysis with this procedure, as was demonstrated for *L. lactis *[29] and *Corynebacterium glutamicum *[30]. The second is a solution of 60% MeOH and 70 mM HEPES. This quenching solution was used for *E. coli *[12] and transcriptome analysis of *L. plantarum *[31]. The third quenching solution is a mixture of 60% MeOH and 0.85% (w/v) NaCl and the fourth contains 60% MeOH and 0.85% (w/v) ammonium carbonate (AC) (pH 5.5). The latter two are new formulations which should both avoid an osmotic shock during quenching without the addition of compounds disturbing post-extraction analysis. The addition of ammonium carbonate has an additional advantage that it is easily removed during freeze drying by evaporation. Since chromatographic separation coupled to mass spectrometry detection is currently the most versatile and suitable analytical method for metabolome analyses [10,11,15,32], the quenching solution with ammonium carbonate might be very appropriate since it avoids the osmotic shock without introducing the typical undesirable ion effects in mass spectrometry.

*L. plantarum *was grown in pH-controlled batch fermentors and subsequently, culture samples were poured into the four different quenching solutions kept at -40°C with a ratio of 1:3 (v:v). After centrifugation, the cell pellets were first washed with the same quenching solution to remove residual extracellular metabolites after which the cell pellets were extracted with perchloric acid. The amount of the intracellular ATP was measured both in the supernatant and in the cell extract for quantitative determination of cell lysis. First, the reproducibility of the quantification method for ATP-release was evaluated. For this, three samples from the same batch culture were quenched with MeOH/HEPES solution. The average percentage of ATP leakage was determined to be 2.5 ± 0.9%. Two samples that were quenched with 60% MeOH showed higher percentages of ATP leakage, with an average of 12.5 ± 0.5% (Table 1). Furthermore, samples from three different batch cultures were quenched with MeOH/HEPES. These showed an average ATP leakage of 4.4 ± 1.5%, which is close to the value obtained with the repetitive sampling of a single batch culture (Table 1). Based on these findings, we consider this method reliable for quantification of the effect of different quenching solutions on the integrity of the cell envelope of *L. plantarum*.

**Table 1:** ATP leakage (%) of different samples from different cultures.

**Samples from same culture**				**Samples from different cultures**	
**Samples**	**MeOH/HEPES**	**Samples**	**MeOH**	**Samples**	**MeOH/HEPES**

1	2.3	5	13.0	7	2.5
2	1.6	6	12.0	8	6.4
3	3.7	-	-	9	4.3
Average	2.5 ± 0.9	Average	12.5 ± 0.5	Average	4.4 ± 1.5

Figures in the table indicate the percentage of ATP that was measured in the supernatant of *L. plantarum *cells after quenching. Samples 1, 2 and 3 come from the same batch culture and were quenched with 70 mM of HEPES (pH 5.5). Samples 7, 8 and 9 come from different batch cultures and were also quenched with MeOH/HEPES solution. Samples 5 and 6 come from the same batch culture but were quenched with 60% aqueous methanol solution. Cultures were grown on CDM with 333 mM and 100 mM of glucose respectively. The percentage was calculated by dividing the amount of ATP in the supernatant by the sum of the amounts of ATP in the supernatants and cell extract.

In non-quenched samples, 5% of the total amount of ATP was found in the supernatant of the batch culture, indicating the base-line level of cell lysis. Less than 10% of the ATP was found in the supernatant after quenching the culture samples with MeOH/HEPES and washing the cell pellets once with the same solution, which is similar to the values observed with *S. cerevisae *cultures that were quenched with aqueous methanol [7]. With MeOH/NaCl, the ATP leakage in *L. plantarum *was found to be higher, while the highest degree of leakage was observed when the cells were quenched with 60% of aqueous MeOH (Table 2).

**Table 2:** ATP leakage (%) with four different quenching procedures.

	**Batch culture**			**Chemostat culture (D = 0.06 h^-1^)**		
**Supernatants**	**MeOH**	**MeOH/HEPES**	**MeOH/NaCl**	**MeOH/HEPES**	**MeOH/NaCl**	**MeOH/AC**

**Quenched**	12.0	6.4	9.5	4.3	15.0	8.1
**1^st ^washed**	14.7	1.2	4.8	0.5	5.4	0.8
**Total**	26.7	7.6	14.3	4.8	20.4	8.9

Percentage of ATP that was measured in the supernatant of *L. plantarum *cells after quenching and washing with different quenching solutions that contained either 60% methanol, 60% methanol and 70 mM of HEPES (pH 5.5), 60% MeOH and 0.85% NaCl or 60% methanol and 0.85% ammonium carbonate (pH 5.5). Samples were taken from batch or continuous cultures, grown on CDM with 333 mM and 100 mM of glucose respectively. The percentage was calculated by dividing the amount of ATP in the supernatant by the sum of the amounts of ATP in the supernatants and cell extract. The average leakage of ATP in the supernatant of non-quenched batch culture samples was 5%.

The different quenching solutions were also applied on chemostat cultures (Table 2). The total ATP loss in the quenching and washing steps due to cell lysis was less than 10% when MeOH/HEPES or MeOH/AC were used. Again the amount of ATP leakage was higher when the culture was quenched with MeOH/NaCl.

These results show that the degree of lysis of *L. plantarum *during quenching depends on the quenching solution that is being used. Ionic strength or pH effects are likely to affect cell lysis, which is below 10% only with MeOH/HEPES or MeOH/AC as quenching solutions.

The second requirement of a reliable quenching method is that the sampling and quenching method is sufficiently rapid to stop the metabolism. For this, the energy charge of quenched samples was determined. *L. plantarum *was grown in a continuous culture on CDM medium with 100 mM of glucose at a D = 0.06 h^-1^. At steady state, 50 mL of the culture, which had a cell density of 1.39 g dry weight/L, was quenched and subsequently washed with the quenching solutions that contained MeOH/HEPES and MeOH/AC. As shown above, the latter two solutions gave the lowest degree of cell lysis. After this, the pellet was extracted with 5 mL of cold methanol. The amounts of ATP, ADP and AMP were determined in the cell extract. Variations between the extracts did not exceed 10% and the energy charge values were found to be 0.83 and 0.70 respectively (Table 3), which is typical for exponential growing cells. In steady state continuous cultures, the growth limiting substrate is only present in very low concentrations, which means that sampling causes a very rapid depletion of substrate in the sample. The high value of the energy charge that was obtained after applying the cold methanol-based quenching procedures indicates that the metabolism of *L. plantarum *was rapidly inactivated. Therefore, the samples can be considered as representative snapshots of the metabolite profile. In contrast, the energy charge of the cells that were sampled from this continuous culture without quenching rapidly dropped since the ATP content was only 16% of the amount of ATP that was present in the cells that were quenched during sampling, indicating that a large amount of ATP was converted during sampling. These results confirm that the energy charge value can function as a critical indicator of the rate of inactivation of the metabolism.

**Table 3:** Adenine nucleotide concentrations and energy charges in cell extracts of a chemostat grown culture of *Lactobacillus plantarum*.

**Quenching solution**	**ATP (mM)**	**ADP (mM)**	**AMP (mM)**	**Total (mM)**	**EC**
MeOH/HEPES	5.88	2.04	0.44	8.36	0.83
MeOH/AC	4.75	2.11	1.53	8.39	0.70

Intracellular concentrations of ATP, ADP and AMP in cell extracts derived from a pH-controlled continuous culture. Two samples were quenched, one with 60% MeOH and 70 mM HEPES (pH 5.5) and the other 60% MeOH and 0.85% ammonium carbonate (pH 5.5). The two cell pellets were washed with the same quenching solution, extracted with cold methanol and freeze-dried. Finally, ATP, ADP and AMP were quantified. The coefficient of variance of this quantification method was less than 7%. The intracellular concentrations were determined using the intracellular volume of *E. coli *(2.15 mL g^-1 ^dry weight) [37].

The energy charge (EC) of the cell extract was calculated by EC=[ATP]+0.5[ADP][ATP]+[ADP]+[AMP]
 MathType@MTEF@5@5@+=feaafiart1ev1aaatCvAUfKttLearuWrP9MDH5MBPbIqV92AaeXatLxBI9gBaebbnrfifHhDYfgasaacH8akY=wiFfYdH8Gipec8Eeeu0xXdbba9frFj0=OqFfea0dXdd9vqai=hGuQ8kuc9pgc9s8qqaq=dirpe0xb9q8qiLsFr0=vr0=vr0dc8meaabaqaciaacaGaaeqabaqabeGadaaakeaacqWGfbqrcqWGdbWqcqGH9aqpdaWcaaqaamaadmaabaGaemyqaeKaemivaqLaemiuaafacaGLBbGaayzxaaGaey4kaSIaeGimaaJaeiOla4IaeGynauZaamWaaeaacqWGbbqqcqWGebarcqWGqbauaiaawUfacaGLDbaaaeaadaWadaqaaiabdgeabjabdsfaujabdcfaqbGaay5waiaaw2faaiabgUcaRmaadmaabaGaemyqaeKaemiraqKaemiuaafacaGLBbGaayzxaaGaey4kaSYaamWaaeaacqWGbbqqcqWGnbqtcqWGqbauaiaawUfacaGLDbaaaaaaaa@4FB9@

### Extraction of metabolites

An ideal extraction agent should extract as many intracellular metabolites as possible with minimal degradation and no enzymatic, chemical or physical modification of the targeted metabolites. However, Mashego and co-workers. [33] concluded that the ambitious goal of quantitative coverage of the cellular metabolome requires development of specific individualized extraction protocols targeting various classes of metabolites. *L. plantarum *cells have been extracted using perchloric acid for sugar analyses [4]. Jensen and co-workers [29] applied chloroform extraction for *Lactococcus lacti*s. To the best of our knowledge, no studies comparing different extraction methodologies tailored for *L. plantarum *have been published. We have tested five different extraction methods for their efficiency on *L. plantarum *cultures. The methods were derived from the procedures described for *E. coli *[22].

The extraction methods that were applied on *L. plantarum *involve permeabilisation by cold methanol, acid treatment (perchloric acid), high-temperature extraction with ethanol and lysis with chloroform/methanol and chloroform/water. The extraction efficiency of each method was determined by measuring the concentrations of ATP, NAD^+^, and G-6P as representatives of different groups of metabolites with different chemical properties, and a high sensitivity to enzymatic conversions.

Cells derived from a batch culture were washed, aliquoted, centrifuged and stored at -80°C until extraction. Triplicate extracts were made for each method and the concentrations of ATP, NAD^+ ^and G-6P were determined (Table 4.A.). The hot ethanol and perchloric acid treatments gave the highest recoveries for all three compounds, while the methods using chloroform resulted in a large decrease of the amount of ATP that could be extracted from the cells. Possible explanations for this observation are (i) the low extraction efficiency or (ii) remaining ATPase activity. The latter could be reduced by the addition of EDTA [7], but this also introduces difficulties for the subsequent metabolite analyses. The procedure that uses cold methanol resulted in the poorest extractability for all 3 metabolites. This result is in sharp contrast to the data of Maharjan and Ferenci [22], who recommended cold methanol as the most suitable extraction agent for global metabolite analysis.

**Table 4:** Intracellular concentrations of ATP, NAD^+ ^and G-6P in *L. plantarum *cells that were extracted with different methods.

**Extraction methods**		**ATP (mM)**	**NAD^+ ^(mM)**	**G-6P (mM)**
A	Cold MeOH	0.05 ± 0.01	8.4 ± 0.6	10.2 ± 0.8
	Perchloric acid	1.07 ± 0.19	13.9 ± 1.2	13.2 ± 1.1
	Hot EtOH	1.15 ± 0.07	14.7 ± 0.9	16.4 ± 0.6
	CHCl_3_/H_2_O	0.49 ± 0.08	13.9 ± 0.4	15.2 ± 0.6
	CHCl_3_/MeOH	0.15 ± 0.02	8.6 ± 0.9	11.4 ± 0.3

B	Modified Cold MeOH	1.53 ± 0.15	11.8 ± 0.4	10.5 ± 0.7
	Perchloric acid	1.05 ± 0.01	11.5 ± 2.2	11.0 ± 1.3
	Hot EtOH	1.38 ± 0.34	9.0 ± 0.1	9.4 ± 1.0

Intracellular pools of ATP, NAD^+ ^and G-6P in *L. plantarum *cells that were extracted with five different methods. These amounts and their relative standard deviation are from different extracts. The cells were harvested without quenching, washed, concentrated. Each sample in the top part of the table (A section) contained 9.8 mg of dry weight and came from 78 mL (dry weight 0.38 g). Samples in the bottom part of the table (B section) contained 8.3 mg of dry weight and came from 100 mL (dry weight 0.27 g) of batch cultures growing on CDM with 333 mM glucose. The intracellular volume was calculated using the intracellular volume of *E. coli *(2.15 mL g^-1 ^dry weight) [37].

During the latter procedure, the cells are first resuspended in cold water before the addition of the cold (absolute) methanol. In this period, the enzymes are not yet inactivated, which might explain the relatively low concentrations of the 3 metabolites. Therefore, a new extraction procedure for the cold methanol treatment was designed in which the *L. plantarum *cell pellet was directly resuspended in absolute cold methanol (-80°C) instead of first using cold water. This adjusted method was compared with the hot ethanol and perchloric acid method using a *L. plantarum *batch culture. As presented in Table 4.B, the metabolite recovery with the cold methanol procedure was improved and was similar to the perchloric acid and hot ethanol extractions. This modification of the cold methanol procedure was crucial for obtaining high retention of the metabolites in the sample fluid. Due to its high extraction efficiency and simplicity, this modified cold methanol procedure was selected as the preferred method for the extraction of intracellular metabolites from *L. plantarum*.

High extraction efficiency also implies also good enzyme inactivation. ATP is very rapidly degraded to less energy rich metabolites. Therefore, a fixed amount of ATP (6.1 nmols) was spiked into the cell pellet prior to extraction with cold methanol to determine whether the ATP converting enzymes were completely inhibited during the whole procedure. Next, the samples were extracted and freeze-dried. ATP, ADP and AMP concentrations were determined and compared with a sample without ATP addition (Table 5). After spiking with ATP, 100% of the total amount of adenylate nucleotides was recovered, but only 85% of ATP could be traced back in the sample. The rest was found to be converted mainly into ADP, indicating that the enzymatic activity was not been completely inhibited.

**Table 5:** Recovery of adenine nucleotides after spiking extracts with a known amount of ATP.

	**Sample (nmol)**	**Sample + ATP addition (nmol)**
**ATP**	5.3	9.7
**ADP**	22.8	24.5
**AMP**	32.3	32.7
**Total**	60.4	66.9

Amounts of ATP, ADP and AMP that were determined in the cell extract before and after the addition of 6.1 nmoles of ATP. The relative standard deviation for the ATP quantifications was less than 5%. The sample was taken from a batch culture that was growing on CDM with 333 mM of glucose without quenching and the cell pellet was extracted with cold methanol. The energy charge value calculated from the energetic metabolites is 0.28 ± 0.01.

## Conclusion

The metabolism of *L. plantarum *cells is quenched efficiently with cold MeOH 60% containing 70 mM HEPES (pH 5.5) or cold MeOH 60% with 0.85% ammonium carbonate (pH 5.5). These procedures result in less than 10% cell lysis. The energy charge value was demonstrated to be a useful indicator for the rate of inactivation of the metabolism of the cell. In contrast to MEOH/HEPES, the new quenching solution MeOH/AC offers the advantage that it will not disturb further metabolite analysis because all components are being removed from the sample during freeze-drying. This study has also demonstrated that the modified cold methanol extraction methodology yields the highest extraction efficiency of the 3 metabolites of choice. Direct extraction of the cell pellet with cold methanol was found to be critical for the extraction efficiency of the method. Moreover, the method does not expose the samples to high temperatures or extreme pH values, and the subsequent metabolite analysis is not hampered by the introduction of extra salts. The only drawback of the method is that enzymatic activity is probably not completely eliminated. Therefore, factors like handling time and temperature should be carefully controlled during the procedure. Our study confirms one of the conclusions drawn by Mashego and co-workers [33] that it is essential to develop tailor-made quenching and extraction methods for each microbial species of interest.

## Methods

### Microorganism and culture conditions

*L. plantarum *WCFS1 was grown in a 1.7 L bioreactor with a working volume of 1 L (Applikon, The Netherlands) on Chemically Defined Medium (CDM) [34] supplemented with glucose as substrate. The culture was stirred with a mechanical stirrer at 200 rpm and kept anoxic by flushing the headspace with nitrogen gas. The temperature was kept at 37°C and the pH was maintained at 5.5 by the automatic addition of 2 M NaOH. Continuous cultivations were performed on CDM medium supplemented with 100 mM of glucose at a dilution rate of 0.06 h-1. Steady state was assumed after 5 volume changes.

### Quenching and extraction procedures

The metabolism of a culture sample was rapidly inactivated by mixing 1 volume of culture sample with 3 volumes of different quenching solutions at -40°C. Four different quenching solutions were used and compared that contained either 60% MeOH, 60% MeOH and 70 mM HEPES (pH 5.5), 60% MeOH and 0.85% (w/v) NaCl, or 60% MeOH and 0.85% (w/v) ammonium carbonate (pH 5.5). After quenching, the cells were kept at -40°C for 30 min, centrifuged (5 min, 3000 g) with a pre-cooled rotor of -40°C and washed with the same volume of quenching buffer. During the whole procedure, the temperature of the samples was kept below -10°C. The supernatants were diluted with the same volume of cold water, freeze-dried, and stored at -80°C until further analysis.

Different methods for the extraction of metabolites from the cell pellets were used and compared based on the procedures described by Maharjan and Ferenci [22]:

- Cold methanol extraction. In the initial experiments, the cell pellet was resuspended in 0.25 mL of ice cold water, after which 0.25 mL of cold methanol (-80°C) was immediately added to the suspension. After vigorously mixing, the suspension was frozen in liquid nitrogen and stored at -80°C for 1 night. In the optimised protocol, the cell pellet was directly resuspended in 1 mL of cold absolute methanol and frozen. Next, the sample was thawed on ice and immediately centrifuged (10000 x g) for 2 min at maximum speed at 4°C. The supernatant was subsequently transferred to a new tube and the extracted pellet was re-extracted twice with 0.5 mL of cold methanol and twice with 0.5 mL of cold water. All extracts were combined and diluted with an equal volume of cold water, after which the solution was frozen in liquid nitrogen, freeze-dried, and stored at -80°C until further analysis.

- Perchloric acid extraction. A 1 mL aliquot of 35% of perchloric acid (-20°C) was added to the cell pellet. After vigorously mixing, the sample was frozen at -80°C and stored overnight. After thawing and centrifugation, the pellet was extracted twice with 0.5 mL of water, and the supernatants were pooled. The supernatant was neutralized by the addition of 100 μL of 2 M of phosphate buffer (pH 7.0) and addition of 5 M KOH. The precipitated KClO4 salt were removed by centrifugation and washed with cold water. The supernatants were frozen in liquid nitrogen, freeze-dried, and stored at -80°C until further analysis.

- Chloroform/water extraction. The pellet was resuspended in 0.25 mL of ice-cold water after which, 1 mL of chloroform (-80°C) was added to the suspension. After vigorously mixing, the sample was incubated at -20°C for 1 day and vortexed for 1 min every 2–3 h. Then, the suspension was centrifuged, and the water phase was transferred to a new tube. The organic solvent phase was washed twice with 0.5 mL of water, after which all water layers were combined and centrifuged to remove the cell debris, frozen in liquid nitrogen, freeze-dried, and stored at -80°C until further analysis.

- Chloroform/methanol extraction. The pellet was resuspended in 0.25 mL of methanol, after which 1 mL of chloroform (-80°C) was immediately added to the suspension. After vigorously mixing, the sample was incubated at -20°C for 1 day and vortexed several times during this period to ensure good interaction between the chloroform and water phase. Then, the suspension was centrifuged, and the pellet was washed with 0.5 mL of methanol. The supernatants were combined and the chloroform was eliminated from the sample by flushing with nitrogen. Finally, 2 mL of cold water was added, after which the sample was frozen in liquid nitrogen, freeze-dried, and stored at -80°C until further analysis.

- Hot ethanol extraction. In the initial experiments, the cell pellet was resuspended in 0.25 mL icecold water, after which 500 μL of boiling ethanol was immediately added to the suspension. In the optimised protocol, 500 μL of boiling ethanol was directly added to the cell pellet. Next, the cell suspension was placed into a hot water bath (90°C) for 10 min, during which the suspension was briefly vortexed twice. After this, the sample was cooled on ice for 3 min and stored at -80°C for 1 night. Finally, the sample was centrifuged and the cell pellet was washed twice with 0.5 mL of water. The supernatants were combined and an equal volume of water was added, after which the sample was frozen.

### Metabolite analysis

The freeze-dried samples were resuspended in 1–2 mL of water and centrifuged (2 min, maximum speed, 4°C), after which the supernatants were neutralized with KOH, and analyzed for metabolites. ATP was directly determined from the luminescence produced in the luciferin-luciferase reaction using the ATP bioluminescence assay kit CLS II (Roche Applied Science, Germany). ADP and AMP concentrations were determined in 100 mM of triethanolamine buffer, pH 7.8 with 30 mM MgSO_4_ and 200 mM KCl based on the procedures described by Bergmeyer et al. [35]. For the ADP analysis, 1.5 mM of phosphoenolpyruvate and 10 μg pyruvate kinase were added. The reaction mixture was incubated for 3 h at 30°C, after which the total amount of ATP was determined. For the AMP analysis, 1.5 mM of phosphoenolpyruvate, 10 μg of pyruvate kinase, and 2.6 μg of myokinase were added to the assay mixture. The mixture was incubated for 3 h at 30°C, after which the total amount of ATP was determined. The amounts of ADP and AMP in the sample were calculated from the increase in ATP concentration.

NAD+ and glucose-6-phosphate (G-6P) were measured using fluorimetric analysis described by Garrigues et al. [36]. Emission was measured at 456 nm (slit 5 nm) after excitation at 350 nm (slit 2.5 nm) with the Safire fluorescence multiplate spectrophometer (Tecan, Switzerland). NAD+ was determined in 250 mM of pyrophosphate buffer (pH 8.8) containing 12 g/L of semicarbazide, 5% (v/v) of absolute ethanol, and 58 μg of alcohol dehydrogenase. G-6P was measured in 100 mM of triethanolamine buffer (pH 7.6) supplemented with 3 mM MgSO4 and 0.8 mM EDTA, 1 mM NAD+ and 2 U of G-6P dehydrogenase.

## Competing interests

The author(s) declare that they have no competing interests.

## Authors' contributions

MF performed all the quenching and extraction experiments as well as the metabolite analysis. AEM performed the fermentations and was involved in the experimental design. EJS participated in the design of this study as well as coordination and supervision of the work. All authors read and approved the final manuscript.
